# Peripheral Neutrophil Functions and Cell Signalling in Crohn`s Disease

**DOI:** 10.1371/journal.pone.0084521

**Published:** 2013-12-19

**Authors:** Rajesh Somasundaram, Veerle J. A. A. Nuij, C. Janneke van der Woude, Ernst J. Kuipers, Maikel P. Peppelenbosch, Gwenny M. Fuhler

**Affiliations:** Department of Gastroenterology and Hepatology, Erasmus University Medical Center, Rotterdam, Rotterdam, The Netherlands; University of San Francisco, United States of America

## Abstract

The role of the innate immunity in the pathogenesis of Crohn’s disease (CD), an inflammatory bowel disease, is a subject of increasing interest. Neutrophils (PMN) are key members of the innate immune system which migrate to sites of bacterial infection and initiate the defence against microbes by producing reactive oxygen species (ROS), before undergoing apoptosis. It is believed that impaired innate immune responses contribute to CD, but it is as yet unclear whether intrinsic defects in PMN signal transduction and corresponding function are present in patients with quiescent disease. We isolated peripheral blood PMN from CD patients in remission and healthy controls (HC), and characterised migration, bacterial uptake and killing, ROS production and cell death signalling. Whereas IL8-induced migration and signalling were normal in CD, trans-epithelial migration was significantly impaired. Uptake and killing of *E. coli* were normal. However, an increased ROS production was observed in CD PMN after stimulation with the bacterial peptide analogue fMLP, which was mirrored by an increased fMLP-triggered ERK and AKT signal activation. Interestingly, cleavage of caspase-3 and caspase-8 during GMCSF-induced rescue from cell-death was decreased in CD neutrophils, but a reduced survival signal emanating from STAT3 and AKT pathways was concomitantly observed, resulting in a similar percentage of end stage apoptotic PMN in CD patients and HC. *In toto*, these data show a disturbed signal transduction activation and functionality in peripheral blood PMN from patients with quiescent CD, which point toward an intrinsic defect in innate immunity in these patients.

## Introduction

Crohn`s disease (CD) is a chronic inflammatory bowel disease with a complex aetiology involving genetic factors, priming by enteric microflora, environmental factors and an alteration in the immune-mediated response [1-3]. Increasing evidence points towards a role of the innate immune system in CD pathology, with a role for dendritic cells, macrophages and neutrophils [4,5]. Neutrophils (polymorphonuclear cells; PMN), one of the most abundant and important mediators of innate immunity, are professional phagocytes which mount the acute inflammatory response and act as the first line of defence against invading pathogens [6]. The role of PMN in CD pathology remains obscure. Impaired PMN function may result in limited bacterial clearance and fuel an on-going, chronic inflammatory response. Indeed, patients with congenital disorders of PMN function (i.e. migration, production of reactive oxygen species [ROS]) often develop inflammatory bowel disease (IBD) [7-10]. Furthermore, mice lacking the NADPH oxidase gene encoding p40^phox^ show enhanced colitis [11] supporting a positive role for ROS in the resolution of disease. On the other hand, epithelial cell damage and ensuing bacterial invasion and inflammation have been attributed to noxious ROS released by PMN, and PMN ablation has proven beneficial in a subset of CD patients [12-14]. Relatively few studies have investigated PMN cell biology in CD, and those that have, show conflicting results. Although an inadequate PMN influx and subsequent clearance of bacteria has been observed in CD, this may be caused by defective secretion of pro-inflammatory cytokines by macrophages, and it is as yet unclear whether PMN intrinsically lack migratory capacity, ROS production or bactericidal activity [15-20]. 

Altogether, varying predictions have been made regarding the role of PMN in the pathogenesis of CD. Recently, a comprehensive analysis of peripheral blood monocytes in patients with quiescent CD revealed intrinsic defects in this cell-type, prior to inflammation and their recruitment to the mucosa [21]. Impaired cytokine profiles were observed in CD monocytes, whereas migration, ROS production and phagocytosis were unaffected. However, an exhaustive analysis of multiple PMN effector functions and the signalling events involved in one study has so far not been conducted but is urgently needed to complement our insight into the innate immune system functionality in IBD patients. In the current study, we investigated whether PMN from quiescent CD patients are constitutively defective, by investigating the capacity of PMN to respond to stimuli inducing migration, phagocytosis, bacterial killing, ROS production and apoptosis, and the correlation thereof to the activity of the signal transduction pathways involved. We show that transepithelial migration and fMLP-induced ROS production as well as fMLP and granulocyte-macrophage colony-stimulating factor (GMCSF)-mediated signalling are altered in CD PMN, whereas phagocytosis and bacterial killing are normal. 

## Materials and Methods

### Patients

This study was approved by the ethical board of the Erasmus MC, Rotterdam, The Netherlands (protocol MEC-2004-168). Patients and healthy controls were included after written informed consent was obtained. In total, 53 patients and 20 healthy controls were included (Table 1). Due to the limited number of PMN obtained from 20 ml of peripheral blood, the ethical limit in our protocol, as well as logistical arrangements, not all the experiments could be performed with the same set of patients. However, the characteristics of the patients used were similar between experiments, thus precluding the skewing of results of secondary reasons such as age or medication. Patients were in clinical remission (quiescent disease) at the time of blood collection, with no evidence of inflammation in endoscopies performed around this time. All experiments on CD PMN were performed simultaneously on PMN from a healthy volunteer. 

**Table 1:** Characteristics of Crohn’s disease patients and healthy controls.

	**Crohn’s disease**	**Controls**
Number, n	53	20
Mean age, yr (range)	38 (20-68)	32 (24-56)
Sex, n (%)		
- female	26 (49%)	5 (25%)
- male	27 (51%)	15 (75%)
Mean age at diagnosis, yr (range)	24 (13-59)	-
Mean duration of disease, yr (range)	13 (1-37)	-
Location, n (%)		
- terminal ileum (L1)	13 (24.5%)	-
- colon (L2)	11 (20.8%)	-
- ileocolonic (L3)	28 (52.8%)	-
- upper GI tract (L4)	1 (1.9%)	-
Fistulising disease, n (%)	21 (39%)	-
Medication, n (%)		
- none	10 (18.8%)	20 (100%)
- Mesalazine	7 (7.6%)	0 (0%)
- Steroids	10 (18.8%)	0 (0%)
- immunosuppressants	14 (26.4%)	0 (0%)
- anti-TNF	25 (47.1%)	0 (0%)

### Granulocyte isolation from human peripheral blood

Heparin anti-coagulated blood was obtained from CD patients and HCs in parallel. Neutrophils were isolated as described previously [22]. Briefly, mononuclear cells were removed by centrifugation of heparinized blood over Ficoll-Paque (Amersham), followed by erythrocyte lysis with ice-cold NH_4_Cl solution. PMN were allowed to recover for 30 minutes at 37°C in RPMI 1640 supplemented with 0.5% human serum albumin (HSA; Sanquin, the Netherlands). PMN were resuspended in incubation buffer (20mM HEPES, 132mM Nacl, 6mM KCL, 1mM MgSO_4,_ 1.2mM KH_2_PO_4_, 5mM glucose, 1mM CaCl_2_ and 0.5% HSA) before they were subjected to functional assays.

### Migration assay

The migration assay was performed using a microchamber transwell system with 3µM pores (Becton Dickinson). PMN (2 x 10^5^) were applied to the upper well of the chamber. Migration was induced by 20 ng/ml IL8 (Peprotech, Rockyhill, NJ) present in the lower compartment of the chamber for 4 hours at 37°C. Basal to apical migration assay was performed using inverted monolayers of Caco_2_ cells, which were grown inverted on collagen-coated transwell inserts for 5 days in DMEM (PAA laboratories, Pasching, Austria)/10% fetal calf serum (FCS, PAA)/ 10ug/ml Penicillin/Streptomycin (Gibco) (37°C and 5% CO2). Confluence of the epithelial cell monolayer was confirmed by testing their permeability to bovine serum albumin (BSA) as described previously [23]. PMN migration was determined by fluorescence-activated cell sorting (FACS) analysis as described, using FACSCantoII (BD Biosciences) [24], and cells migrated towards IL8 were expressed as percentage of those migrated in control wells without IL8. 

### Phagocytosis and bactericidal activity of PMN

Bacterial uptake and killing were performed as previously described [25]. Briefly, *E. coli* bacteria, transformed with GFP expression vector were grown in kanamycin-containing LB media until OD of 1, after which cultures were centrifuged and resuspended in 1ml of PBS supplemented with 0.1% Gelatin and 10mM HEPES. Bacterial opsonisation was carried out by incubating bacteria with non-heat inactivated human serum (Gibco) for 15 minutes at 37°C. PMN were challenged with 100 µl of opsonised bacteria at 37°C for 15 minutes, using 0°C control for each experiment. The percentage of phagocytosing PMN, as well as their fluorescence intensity as a measure of the amount of phagocytosed bacteria, were determined by flow cytometry. Bacterial killing was tested by washing *E. coli*-challenged PMN 2 times, and resuspending the cell pellet in 1ml of antibiotics-containing buffer in order to kill any contaminating bacteria attached to the plastic. Bacterial killing was allowed to take place for 4 hours. PMN were lysed using sterile water, lysates were plated on LB agar plates and the colonies grown after 18 hours were counted using a colony counter. Each experiment was done in duplicate. 

### ROS production assay

ROS production was performed as previously described [26]. Briefly, PMN (2x10^6^cells/ml) were incubated with DHR123 (Sigma-Aldrich) for 15 minutes and stimulated with 1µM fMLP (Sigma-Aldrich) for 30 minutes. For priming experiments, cells were pre-treated with 5ng/ml GMCSF (Sargramostim, Bayer, Germany) for 15 minutes prior to fMLP stimulation. Stimulation was terminated by washing the cells with ice-cold PBS containing 1% HSA and placing them on ice. Oxidation of DHR123 to the fluorescent Rhodamine 123 was measured by flow cytometry within 30 minutes of termination of stimulation. 

### Apoptosis analysis

Apoptosis was induced by culturing PMN (2x10^6^/ml) with anti-Fas antibody (Fas-Ab, CH 11, 100ng/ml, Millipore). Alternatively, PMN were treated with GMCSF (10ng/ml). After 6 hours of Fas-Ab-induced apoptosis and 15 hours of GMCSF-induced rescue, the percentage of apoptotic PMN was measured by Annexin-V kit according to the manufacturer’s instructions (BD Biosciences, San Jose, CA). Necrotic PMN were excluded by 7AAD (BD Biosciences, San Jose, CA) positivity. Late apoptosis was measured by internucleosomal DNA fragmentation using Apo direct *in situ* DNA fragmentation assay kit (Biovision, Milpitas, California). Briefly, this TUNEL-based detection kit utilizes terminal deoxynucleotidyl transferase (TdT) to catalyse the incorporation of fluorescein-1 2-dUTP at the free 3`-hydroxyl ends of the fragmented DNA. Stained PMN were analyzed using Flowcytometry and the data were analyzed using FlowJo software (Ashland, OR). 

### Quantitative western blot analysis

**Figure 1:**
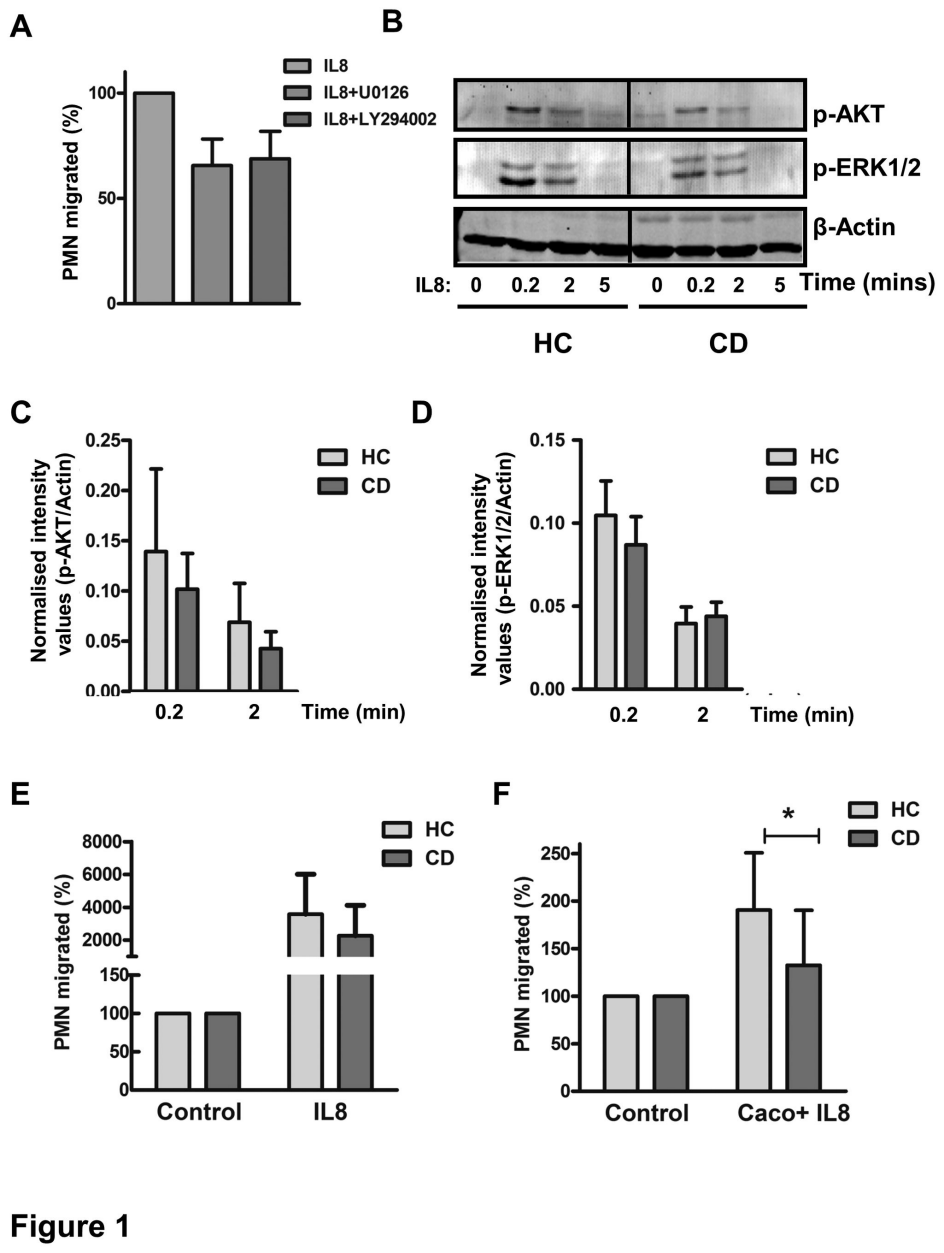
PMN from CD patients are deficient in trans-epithelial migration towards IL8. (A) The involvement of ERK1/2 and PI3K pathways in IL8-induced migration was confirmed by measuring the percentage of migrated PMN after incubation with or without 10 µM of U0126 and LY294002, respectively. Mean±SEM is shown (n=3). (B) PMN were stimulated with 20 ng/ml of IL8 for the indicated time points. Experiments were performed on healthy controls (HC) and CD PMN simultaneously, and samples were loaded side-by-side on the same gel. ERK1/2 and AKT activation were detected by their phospho-specific antibodies. Representative example is shown. (C) No differences in levels of activated ERK1/2 were observed between CD patients and HC (n=10, mean±SEM shown) upon quantification of blots by densitometry. (D) No differences in levels of activated AKT were observed between CD patients and HC (n=10, mean±SEM shown) upon quantification of blots by densitometry. (E) PMN from HC and CD patients were applied to the upper compartment of a transwell system. PMN transmigrated in response to 20 ng/ml IL8 present in the lower compartment were counted by flow cytometry and results are represented as percentage of those migrated in control wells. No differences were observed between Mean±SEM of CD patients (n=11) and HC (n=8). (F) PMN from healthy and CD patients were allowed to migrate through a monolayer of epithelial cells towards IL8 for 4 hours at 37°C. Compared to HC PMN, CD PMN showed significantly less migration (Mean±SEM, *p=0.02, n=10).

PMN were stimulated with 1µM fMLP, 5ng/ml GMCSF, 5ng/ml GCSF or 100ng/ml Fas-Ab (CH 11) as indicated in the figures. Pelleted cells were resuspended in Laemmli buffer, boiled, separated by SDS-PAGE and electrophoretically transferred to PVDF Immobilon FL membrane (Milipore, Billerica, MA). Membranes were probed with antibodies against phospho-ERK1/2 (Thr202/Tyr204), phospho-AKT (Ser473), phospho-STAT3, Caspase 3 (cleaved and uncleaved) or cleaved Caspase 8, all from Cell signalling technology (Danvers, MA). Total levels of ERK, AKT and STAT3 are unaffected by short term stimulation of cells with IL8, fMLP or GMCSF [27-31]. In addition, we demonstrated that total levels of these proteins show excellent correlation with total β-actin levels (Figure 1). Therefore, equal loading was confirmed by reprobing blots with antibodies against β-actin (Santa Cruz Biotechnology, Santa Cruz, CA) according to the manufacturers’ protocols. Proteins were detected by IR dyes (LI-COR, Lincoln, NE). Quantification of phosphorylation and cleavage of Caspases were performed by densitometry of the images, using Odyssey 3.0 software. 

### Statistical analysis

Comparisons between CD and HC samples were tested by non-parametric test for unpaired samples (Mann-Whitney testing) in functional experiments. For western blot analysis, where CD and HC samples were paired per gel, comparisons for paired samples were tested by Student-T-test using Graphpad software (La Jolla, CA).

## Results

### Decreased trans-epithelial migration of neutrophils from CD patients in response to IL8

**Figure 2:**
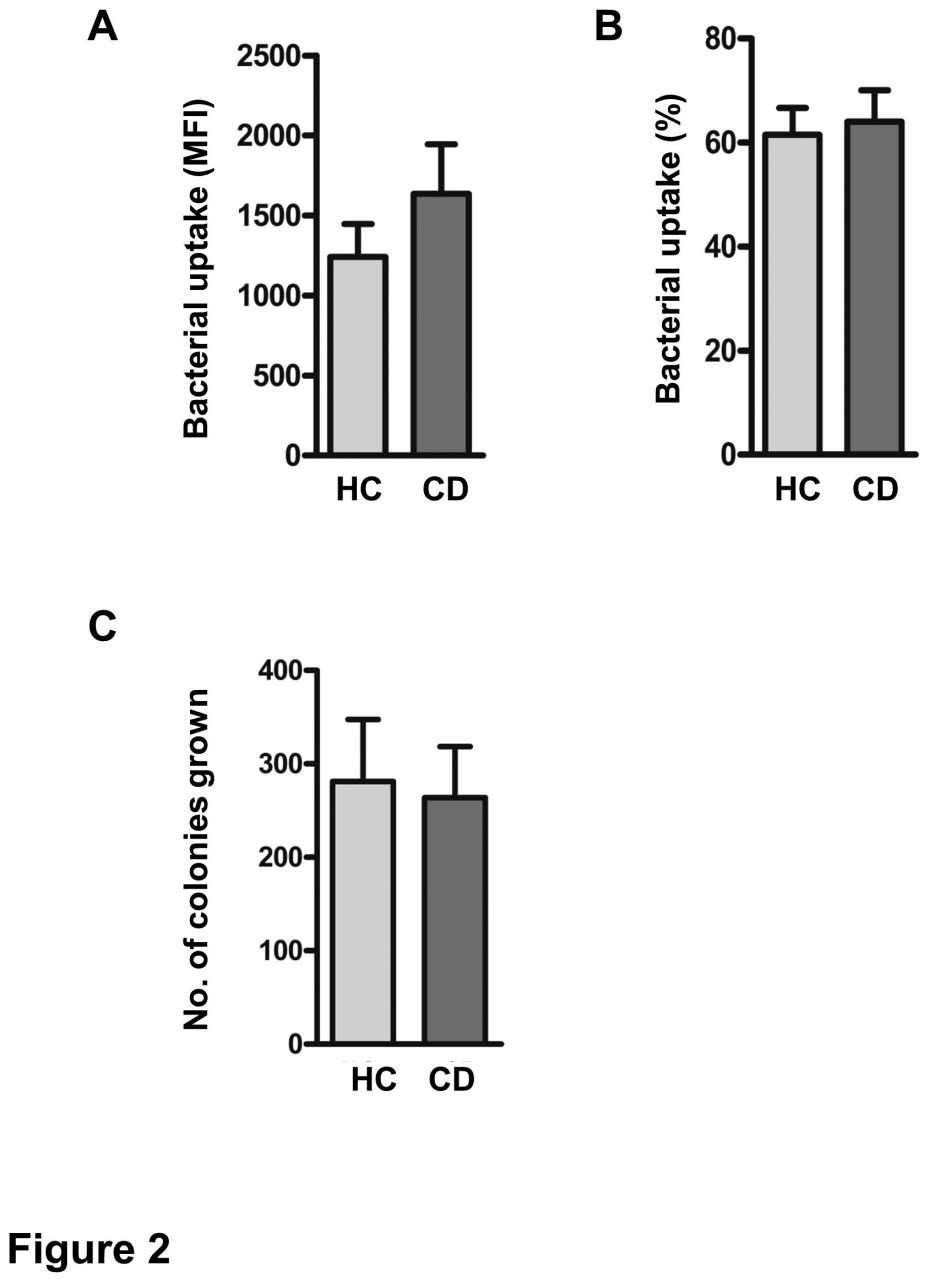
Normal bacterial uptake and killing by PMN from CD patients. Isolated PMN were challenged with opsonised GFP-expressing *E. coli* for 15 minutes at 37°C after which GFP fluorescence was determined by FACs analysis. Appropriate 0 °C control was taken for each experiment. (A) Mean±SEM of median fluorescence intensity (MFI) of PMN from CD patients (n=16) and HC (n=14) is shown. (B) Percentage of PMN positive for *E. coli*–GFP (%) of 16 CD patients and 14 HC. (C) PMN were challenged with *E. coli* for 15 minutes at 37°C and allowed to kill bacteria for 4 hours at 37°C. Colonies grown from lysed PMN after 15 hours were counted using a colony counter. Mean±SEM of CD patients (n=10) and HC (n=9) is shown.

First, we investigated the migratory capacity of CD neutrophils and two of the major signalling pathways involved therein, the ERK1/2 and PI3K-AKT signalling moieties [32]. After confirming the partial dependence of IL8-induced migration on these pathways by using their respective specific inhibitors (Figure 1A: 100 vs. 65.5 ±21% for U0126 and 100 vs. 67 ±22% for LY294002), we examined the phosphorylation of these signal transducers in PMN from CD patients and HCs. We observed a rapid and transient activation of ERK1/2 and AKT in response to IL8 stimulation, but found no significant differences in the level of activation of these molecules between CD patients and HCs (Figure 1B, C and D, n=10). Total levels of ERK were similar between CD patients (n=18) and HC (n=16, p=0.7, Figure 2A and B). In line with this unaltered migration-dependent signalling, the percentage of PMN migrating towards IL8 was not different between CD patients (n=11) and HCs (n=8) (2267±1859% vs. 3574±2443%, p=0.114, Figure 1E). 

In an *in vivo* setting, IL8-mediated migration of PMN towards the lumen of the gut requires basolateral-to-apical migration of PMN over epithelial cells. As this process is substantially differently regulated as compared to migration of PMN toward cytokines alone [33,34], we also determined the level of basolateral-to-apical migration of PMN through an inverted monolayer of human epithelial Caco_2_ cells. Interestingly, the percentage of PMN migrating towards IL8 through epithelial cells was significantly reduced in CD patients compared to HCs (Figure 1F, mean±SEM of 133±55% vs. 190±60%, n=10, p = 0.04). Together, these data suggest that IL8 stimulation of CD PMN in itself results in normal activation of the ERK and PI3K pathways and migration, whereas intrinsic trans-epithelial migration capacity of PMN from CD patients is impaired. 

### Bacterial uptake and killing are not affected in CD patients

Next, we investigated the uptake of GFP-positive *E. coli* by isolated PMN from CD patients (n=16) and HCs (n=14). As shown in Figure 2A and B, neither the percentage of phagocytosing PMN (mean±SEM of 64±24% vs. 62±19%, p=0.7) nor the number of bacteria taken up per granulocyte (1648±1244 vs. 1242±759 MFI, p=0.313) were significantly different between CD patients and HCs. In addition, an equal amount of bacterial colonies were grown from CD and HC PMN, demonstrating that the efficiency of bacterial killing was not different between patients (n=10) and controls (n=9) (263±172 vs. 305±199 colonies, Figure 2C). These results indicate that there is no intrinsic defect in either phagocytosis or killing of *E. coli* bacteria in PMN from CD patients with quiescent disease. 

### Enhanced fMLP-induced ROS production in CD patients, corresponding with increased ERK and AKT signalling

**Figure 3:**
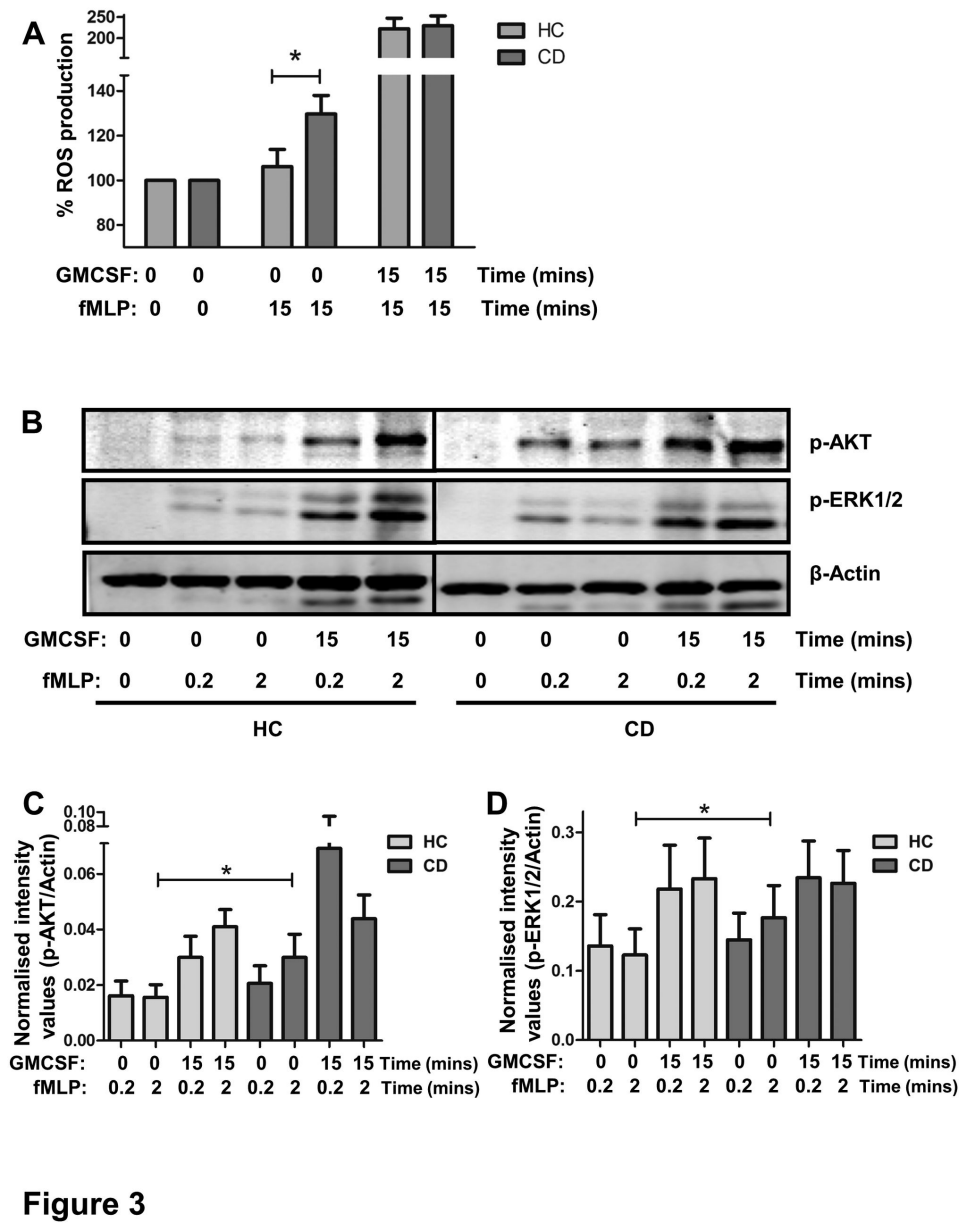
Enhanced fMLP-induced ROS production in CD patients corresponds with increased ERK and AKT signalling. (A) PMN production of superoxide after stimulation was measured by flow cytometry analysis and expressed as a percentage of the fluorescence in unstimulated cells. Mean±SEM of CD patients and HC is shown. Asterisks indicate significantly higher ROS production in fMLP stimulated cells in CD patients compared to HCs (*p=0.03, n=14). Preincubation of PMN with 5ng/ml GMCSF enhanced fMLP-induced ROS production, to an equal maximum in CD patients and healthy controls. (B) Isolated PMN from CD and HC were simultaneously stimulated with 1 µM fMLP with or without priming with 5ng/ml of GMCSF. Phosphorylated ERK1/2 and AKT (upper panels) was detected by Western blot analysis. Membranes were reprobed with antibodies against β-actin (lower panel) to confirm equal loading. (C) Quantification of blots shows that fMLP-induced phosphorylation of AKT is significantly increased in CD patients compared to HC PMN (mean±SEM, *p=0.03, n=9). (D) Quantification of blots shows that fMLP-induced phosphorylation of ERK1/2 is significantly increased in CD patients compared to HC PMN (mean±SEM, *p=0.03, n=9).

The production of ROS is an important antibacterial defence mechanism of PMN. We therefore studied the amount of superoxide produced, and the signalling events involved, in response to the bacterial peptide analogue fMLP. As shown in Figure 3A, fMLP–stimulated ROS production was significantly higher in PMN from CD patients as compared to HCs (mean±SEM of 130±31% vs. 106±28%, p=0.03, n=14). This corresponded to a significantly enhanced fMLP-induced phosphorylation of the ERK and PI3K/AKT pathways (known to be required for ROS production [35]), in PMN from CD patients (p=0.03 and p=0.02, respectively at t=2 min, n=9, Figure 3B-D). These results suggest that PMN from CD patients may already be partially primed *in vivo*. Priming is normally established by pro-inflammatory cytokines such as granulocyte-macrophage colony-stimulating factor (GMCSF), and serves to drastically enhance the respiratory burst in response to bacterial peptides in an inflammatory environment. Indeed, priming of PMN with GMCSF resulted in a significantly higher fMLP-triggered ROS production in both CD and HC PMN (p<0.05). However, ROS production after priming with GMCSF did not differ between CD patients and HCs, indicating that maximal achievable respiratory burst is equal between these groups. As for ROS production, priming of PMN with GMCSF resulted in a significantly enhanced fMLP-triggered phosphorylation of both ERK1/2 and AKT, which again was equal between CD patients and HCs. Similar results were obtained when ROS production and signalling were investigated in GCSF-primed PMN (not shown).

Together, these results suggest that PMN from CD patients release ROS more rapidly in response to bacterial stimuli, but that the maximum achievable level of ROS production is unaltered. 

### Reduced Caspase cleavage during spontaneous apoptosis in PMN from CD patients

**Figure 4:**
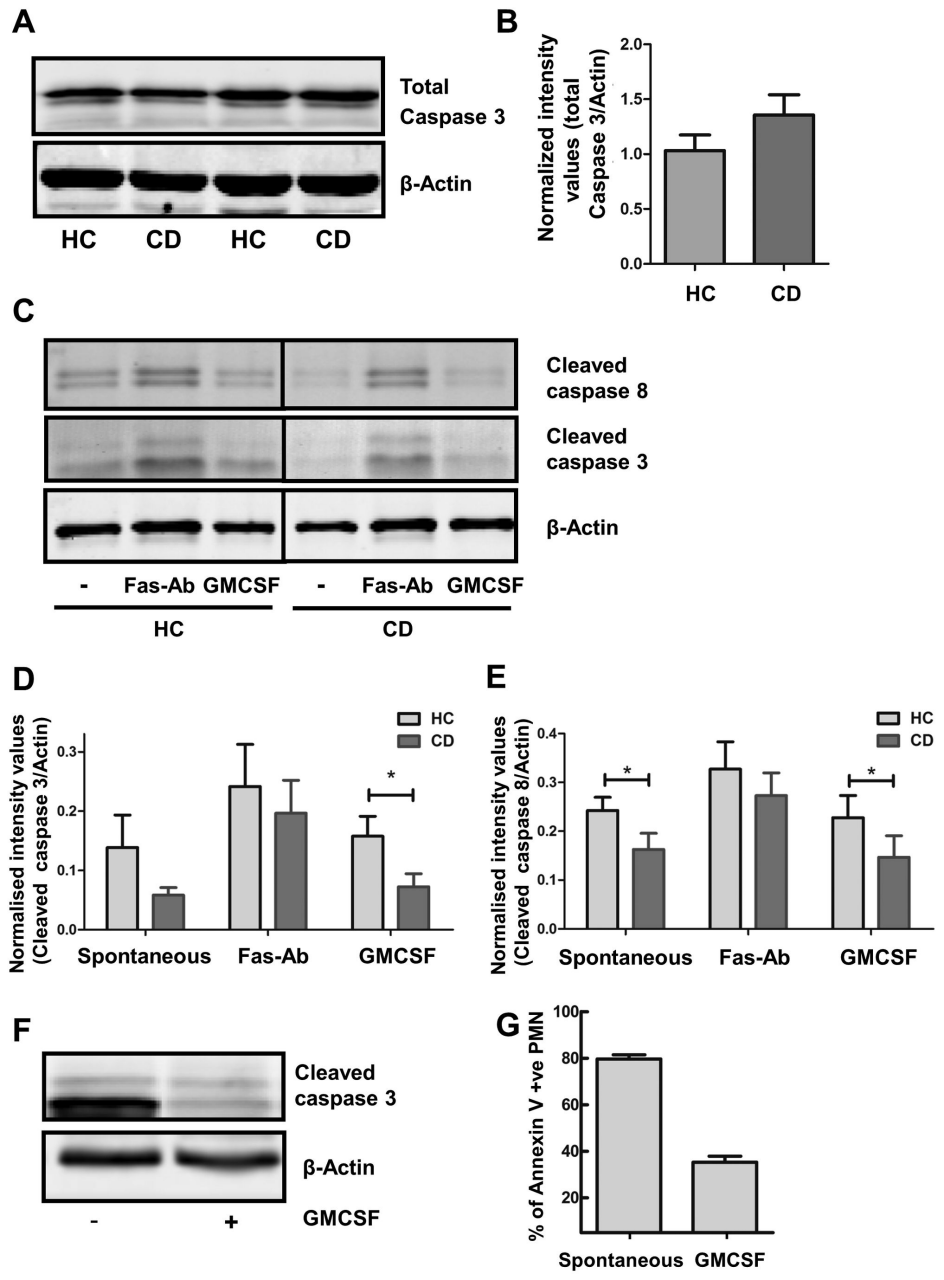
Reduced Caspase cleavage during spontaneous apoptosis in PMN from CD patients. (A) Total Caspase 3 levels were detected in freshly isolated PMN from HC and CD patients by western blotting. A representative example is shown. More examples are shown in Figure 2E. (B) Quantification by densitometry revealed a non-significant increase in total Caspase 3 in PMN from CD patients (n=18) as compared to HC (n=13), p=288. (C) Isolated PMN from CD patient and HC were cultured either with or without 100 ng/ml Fas-Ab (6 hours) or 10 ng/ml GMCSF (15 hours). Samples were loaded onto one gel, and cleavage of Caspase 3 and Caspase 8 were detected by western blotting. Representative example is shown (n=6). (D) Densitometric quantification shows reduced Caspase 3 cleavage in PMN from CD patients undergoing spontaneous apoptosis or GMCSF mediated rescue thereof (mean±SEM, *p=0.04, n=6). (E) Quantification demonstrates reduced Caspase 8 cleavage in PMN from CD patients undergoing spontaneous apoptosis or GMCSF mediated rescue thereof (*p=0.003, n=6). Total Caspase 8 levels were unchanged in CD patients (See Figure 2C and D) (F) PMN were cultured with or without 10 ng/ml GMCSF for 15 hours and rescue of apoptosis by GMCSF was shown by the reduced presence of cleaved Caspase 3, as detected by western blotting (representative example of three independent experiments) (G) Rescue of spontaneous apoptosis after 15 h by GMCSF was detected by quantification of the percentage of apoptotic PMN by Annexin V binding by flow cytometry.

After performing their bactericidal function, PMN undergo apoptosis and are cleared by macrophages. One of the early signalling events to take place in cellular apoptosis is the cleaving of Caspase 8 and 3. Although no differences in total Caspase 3 and Caspase 8 levels were observed in patients (Figure 4A and B, and Figure 2C-E), the amount of Caspase 3 and 8 cleaved during spontaneous cell death was reduced in CD compared to HCs after 6 hours (Figure 4C-E, p=0.1 and p=0.04 for Caspase 3 and Caspase 8 respectively, n=6). Treatment of cells with the apoptosis-inducing Fas-antibody CH 11 enhanced cleavage of both Caspases to an equal extent in patients and HCs (Figure 4C-E, p=0.5 and p=0.2 for Caspase 3 and Caspase 8 respectively, n=6), indicating that only the intrinsic apoptosis machinery is affected in CD. 

Treatment of PMN with GMCSF for 15 hours protects against cleavage of Caspase 3 (Figure 4F), which corresponds to a decreased number of apoptotic cells as measured by externalisation of phosphatidylserine (PS) by Annexin V staining (Figure 4G). When comparing CD patients and HCs for Caspase cleavage in the presence of GMCSF, a significantly enhanced GMSCF-induced survival signal was observed in CD patients, as evidenced by reduced cleavage of Caspase 3 and 8 (Figure 4C, D and E, p=0.04 and p=0.003, respectively, n=6).

### Normal end-stage apoptosis in PMN from CD patients

**Figure 5:**
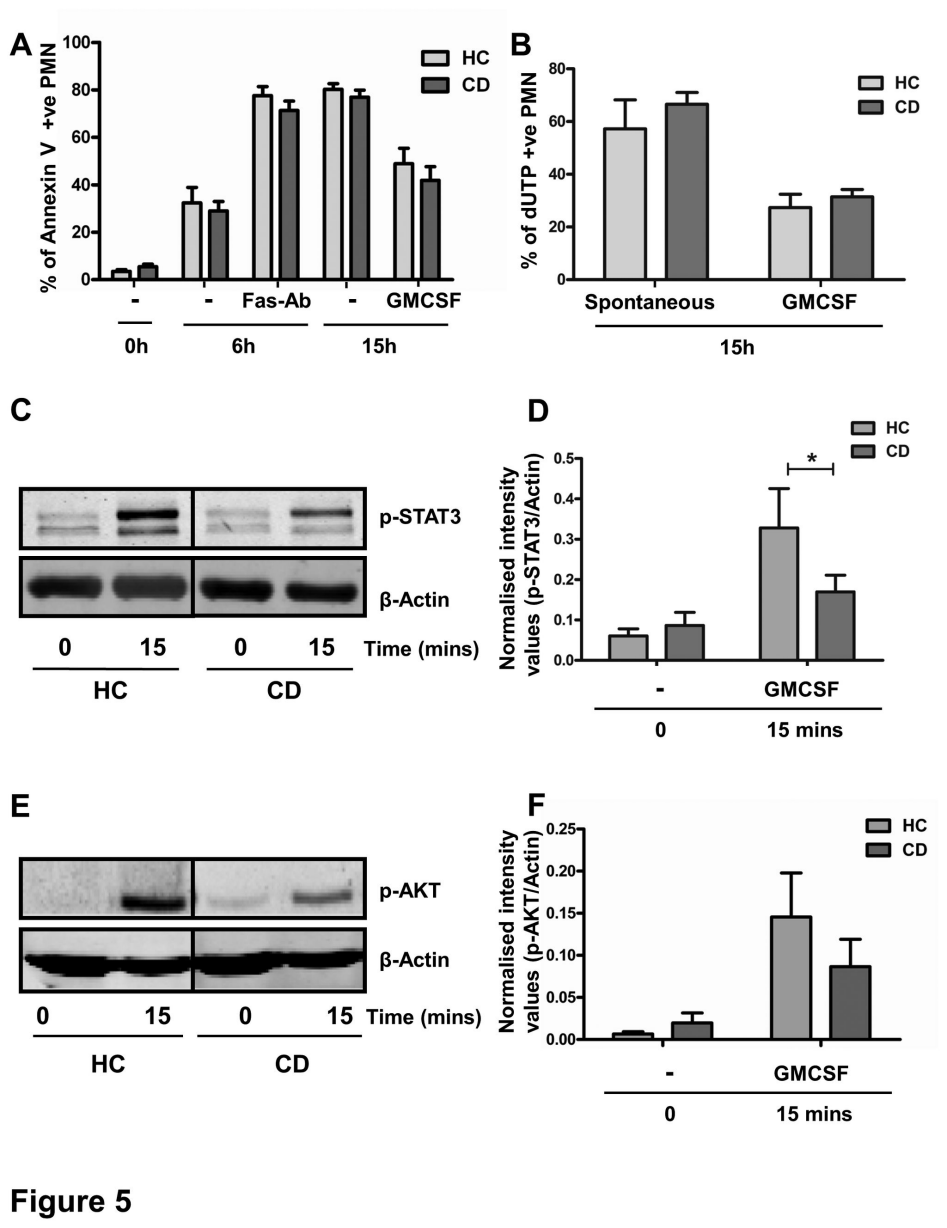
Impaired survival signalling in CD PMN does not affect intermediate and end-stage apoptosis. (A) Isolated PMN were cultured either with or without 100 ng/ml Fas-Ab (6 hours) or 10 ng/ml GMCSF (15 hours) and the percentage of apoptotic PMN was determined by Annexin V positivity (9 CD patients and 8 HC). (B) No differences in DNA fragmentation as measured by TUNEL assay after 15 hours of PMN culture with or without 10 ng/ml GMCSF were observed between CD and HC (n=6). (C-F) PMN were isolated simultaneously from a CD patient and healthy control, stimulated with 5 ng/ml GMCSF for 15 minutes and samples were run on one gel. (C) STAT3 activation was detected by western blotting using phospho-STAT3 antibodies. Representative experiment is shown. (D) Significantly decreased levels of activated STAT3 were observed in CD patients compared to HC (mean±SEM, *p=0.04, n=5). Total STAT3 levels were unchanged (see Figure 2A and F). (E) AKT activation was detected by western blotting using phospho-AKT antibodies Representative experiment is shown. (F) Protein levels of activated AKT were quantified by densitometry and corrected for β-actin protein levels. Mean±SEM of CD patients and HC is shown (n=5).

To test whether the reduced Caspase 3 and 8 signal in CD patients results in a decreased cell death, we measured the percentage of annexin-V-positive cells in PMN cultures at t = 0, 6 and 15h of culture. As expected, cell viability immediately upon isolation (t=0h) was more than 90% (mean±SEM of 5.4±3.6% dead cells in CD, n=9, vs. 3.5±2.2% in HC, n=8, p=0.1, Figure 5A). Surprisingly, spontaneous apoptosis, observed within 6 hours, was not reduced in CD patients compared to healthy controls (mean±SEM of 30±19% vs. 27±11%, p=0.9). Engagement of the Fas-receptor drastically increased the amount of annexin V-positive cells, equally in CD patients and HC (71±12% vs. 77±11%, p=0.2). 

In addition, whereas Caspase cleavage in GMCSF cultured PMN was significantly reduced in CD patients, apoptosis as measured by PS-expression showed no differences between CD and HC PMN in either spontaneous apoptosis after 15h, or the rescue thereof by GMCSF (mean±SEM of 42±17% vs. 48±18%, p=0.7). These findings were confirmed by TUNEL assay, showing no significant differences between CD patients and HCs in the percentage of apoptotic PMN cultured with or without GMCSF for 15 h (mean±SEM of 66±5% vs. 57±20%, p = 0.45 and 31±7% vs. 27±11%, p = 0.49, respectively, n=6, Figure 5B). 

### Decreased GMCSF-induced STAT3 phosphorylation in PMN from CD patients

Whereas GMCSF-induced rescue of Caspase cleavage was enhanced in CD patients, this was not mirrored by an increased survival of PMN. These results suggest that other death mechanisms may override the positive survival signal in CD patients. We therefore investigated GMCSF-induced phosphorylation of STAT3 and AKT, constituting two of the major survival mechanisms induced by this cytokine [31,36]. As shown in Figure 5C and D, STAT3 phosphorylation in response to GMCSF was significantly reduced in PMN from CD patients compared to their healthy counterparts, whereas total STAT3 levels were unchanged (Figure 2A and F). Similarly, a reduced AKT phosphorylation was observed in 4 out of 5 CD patients. These results suggest that an impaired STAT3 and AKT-survival pathway in CD patients may counteract the reduced Caspase cascade activation, thus resulting in equal numbers of apoptotic PMN in CD and HC. 

## Discussion

In the current study, we demonstrate that intrinsic properties of PMN from patients with quiescent CD are changed. A decreased trans-epithelial migration, increased ROS production in response to bacterial peptides, and impaired cellular signalling were observed. Inadequate PMN influx and subsequent clearance of bacteria in CD may contribute to disease status [15,37]. However, conflicting findings are reported regarding intrinsic migratory capacity of PMN in CD patients. *In vitro* migration of PMN from CD patients was found to be normal or increased in transwell assays [16], whereas *in vivo* PMN migration towards skin blisters, skin windows or injured intestinal mucosa was decreased in CD PMN [15,38]. This was attributed to reduced cytokine production, as blister fluid in CD patients contained less IL8, and PMN migration towards skin windows was restored by adding exogenous IL8 [15]. However, increased mucosal IL8 levels have been reported in CD patients, suggesting that other factors may contribute to impaired intestinal PMN migration [39,40]. There are clear differences between migration through bare transwell filters *versus* transepithelial migration [33], with PMN transepithelial migration requiring ICAM-1 and other adhesion-mediated events [41], and epithelial cells producing and releasing a range of chemoattractants at their apical side, which may enhance PMN basolateral-to-apical migration [42]. We now show that *in vitro* transepithelial migration, a clean measure of the intrinsic capacity of PMN to migrate through intestinal epithelial cells, is impaired in CD patients. Whereas IL8-mediated ERK1/2 and AKT signalling is unlikely to contribute to this impairment, a number of adhesion defects may underlie this decreased migration. For instance, IL8 is known to enhance CD11b expression on PMN [43]. Increased expression of the adhesion molecule CD11b on CD PMN has indeed been described, which may be linked to enhanced adhesion and reduced migration in CD [44,45]. Thus, although epithelial cytokine production in CD may be altered, our study shows that an intrinsic defect in PMN transepithelial migration exists, which may contribute in decreased neutrophil recruitment to sites of inflammation. 

Defective bacterial clearance has been associated with the development of CD [46,47] . Loss of NADPH oxidase activity leads to reduced bactericidal activity of PMN, and defective ROS production in a number of inherited disorders is highly associated with intestinal inflammation that is undistinguishable from CD [48]. A recent study by Hayee et al. showed impaired fMLP-induced ROS production in CD PMN, but no defect in bacterial killing [20]. Whereas we confirmed the normal bacterial phagocytosis and killing, our study also demonstrated an enhanced fMLP-induced respiratory burst in CD PMN. As it has long been recognised that oxidative damage plays a major role in mucosal injury in CD, it is conceivable that an exaggerated bacterial peptide-induced PMN ROS production, independent on priming by pro-inflammatory cytokines, may contribute to mucosal damage [12,13]. The discrepancy between these and other studies may be partially explained by differences in study cohorts. Treatment regimens present in CD patients but not HC may have an impact on cellular function. In addition, in our HC cohort, ratio male/female was slightly higher than in the CD group. Although we cannot formally exclude the possibility that this affects results, gender in general does not seem to affect PMN ROS production, migration or phagocytosis [49,50]. In addition, it has been speculated that the genetic alterations associated with increased risk for IBD development, affect innate immune cell function [51]. However, the number of genetic alterations and their method of action on PMN signalling and function is unknown, and is thus difficult to take into consideration in this type of study. The enhanced fMLP-mediated ROS production observed in this study was mirrored by enhanced ERK1/2 and AKT signalling in CD patients, confirming our results, and suggesting that PMN in CD patients may already be primed to some extent *in vivo*. As CD remains incurable, flaring of disease at some point is inevitable, and it is conceivable that circulating levels of pro-inflammatory cytokines are already present even in the absence of a clear inflammation.

PMN are short-lived cells. In the absence of appropriate stimuli, they rapidly undergo characteristic changes indicative of apoptosis. These include cleavage of Caspases, followed by PS exposure on the cell membrane, cleaving of the DNA repair enzyme poly (ADP-ribose) polymerase (PARP), and ending in DNA fragmentation [52]. Delayed PMN apoptosis can result in persisting inflammation and host tissue injury [53]. In this study, we demonstrate a decreased Caspase 3 and 8 cleavage during spontaneous apoptosis and rescue thereof by GMCSF. Surprisingly, this was not mirrored by an enhanced long term PMN survival, as determined by either Annexin V staining or TUNEL staining. *In vivo*, rescue of PMN from apoptosis by GMCSF ensures a longer window of opportunity for PMN to kill invading pathogens, and is mediated through activation of the PI3K/AKT and STAT3 pathways [54,55]. We now demonstrate that both AKT and STAT3 activation are severely reduced in PMN from CD patients in response to GMCSF. It is conceivable that a reduced survival signal coming from these pathways may counteract the enhanced survival mediated through reduced Caspase cleavage. Our data strongly suggest an improper activation of apoptotic signalling pathways in CD PMN, the net result being a normal frequency of apoptotic cells. Whether other functional properties are affected by this impaired signalling remains to be elucidated. 

*In toto*, we demonstrate that intrinsic defects in transepithelial migration, ROS production and chemokine and cytokine induced signalling are present in PMN from quiescent CD patients. CD is a heterogeneous disease, where different underlying mechanisms may cause patient-to-patient variability. Genetic variation is likely to contribute to PMN function, and it is probable that some roles of innate immune cells are underestimated or even obscured by pooling CD patients. Nevertheless, our study clearly shows that genetic variation notwithstanding, several PMN functions are impaired across patients, strongly implying a role for innate immunity in the development of this disease. Through these and other studies, a role for the innate immune system in the development of CD is becoming ever more apparent.

## Supporting Information

Figure S1 **Short term stimulation of PMN does not affect total ERK or STAT3 levels.** Isolated PMN were stimulated with 1 µM fMLP with or without priming with 5ng/ml of GMCSF. Stimulation was confirmed by probing blots with p-AKT or p-STAT3 antibodies (B). Probing blots with total ERK1/2 (A) or STAT3 (B) antibodies showed that stimulation does not hugely influence total protein levels. Moreover, total ERK and total STAT3 protein levels show excellent correlation with β-Actin levels in the same lanes (C and D, respectively), showing that β-Actin is a good loading control.
(TIF)

Figure S2 **No differences in total ERK, STAT3, Caspase 3 or Caspase 8 levels between CD patients and healthy controls (HC).** Unstimulated, isolated PMN from CD patients and HC were run on SDS-PAGE, and probed with antibodies against total ERK protein, total STAT3 protein (examples in panel A), total uncleaved Caspase 8 (panel C) or uncleaved Caspase 3 (examples panel E, more in main manuscript). Quantitation of blots showed no differences in total ERK levels between CD (n=18) and HC (n=16, p=0.6915, panel B). There were no differences in total STAT3 levels between CD (n=24) and HC (n=22, p=0.448, panel F). There were no differences in total uncleaved Caspase 8 levels between CD (n=16) and HC (n=16, p=0.266, panel D). Quantitation of total uncleaved Caspase 3 levels is shown in manuscript, Figure 4B.
(TIF)
