# Supplementary figures and images for: Peripheral Neutrophil Functions and Cell Signalling in Crohn`s Disease
Source: PLoS One. 2013 Dec 19;8(12):e84521. doi: 10.1371/journal.pone.0084521 (PMC3868631; doi:10.1371/journal.pone.0084521)

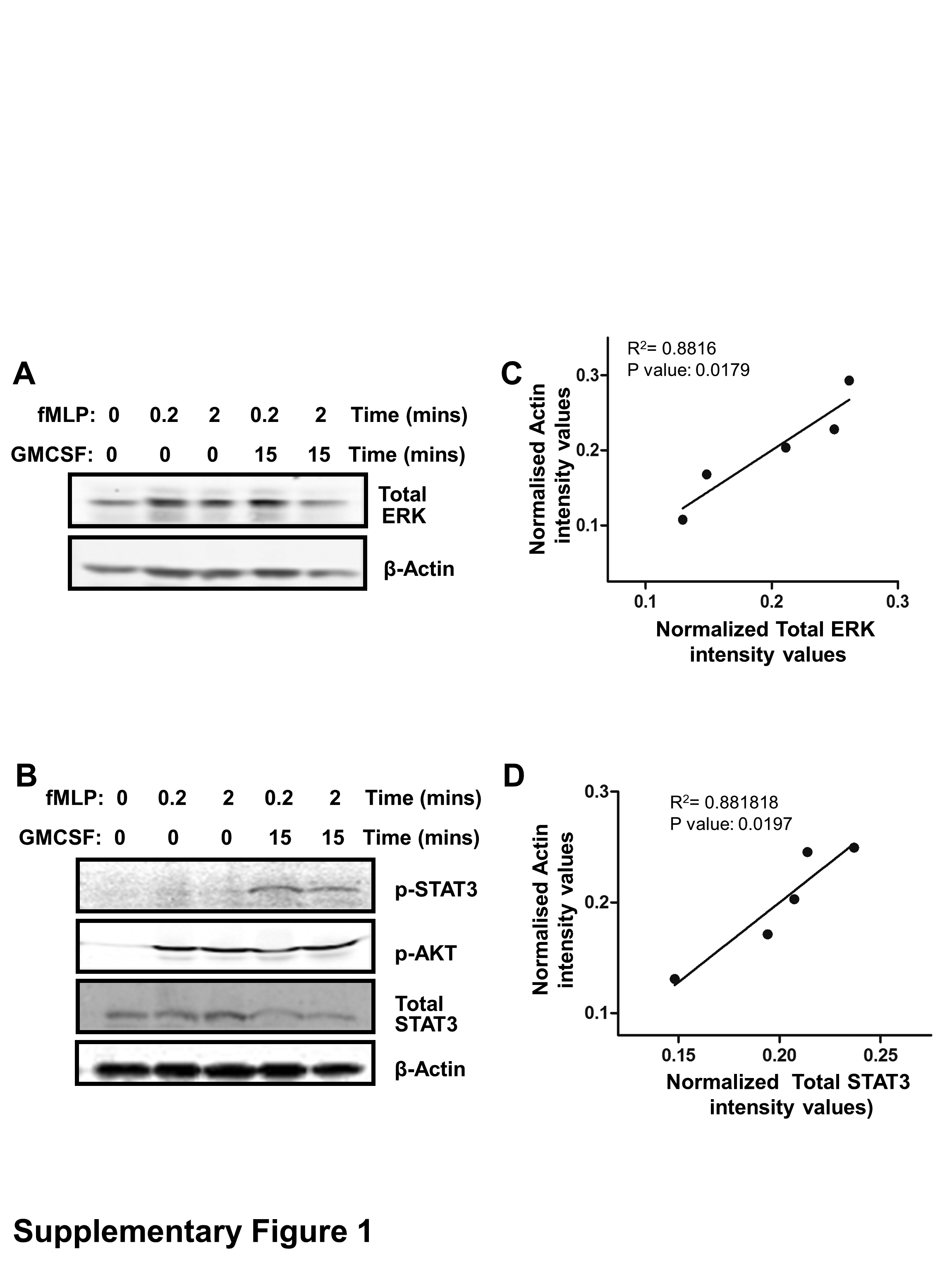

Supplement: Figure S1 — Short term stimulation of PMN does not affect total ERK or STAT3 levels. Isolated PMN were stimulated with 1 µM fMLP with or without priming with 5ng/ml of GMCSF. Stimulation was confirmed by probing blots with p-AKT or p-STAT3 antibodies (B). Probing blots with total ERK1/2 (A) or STAT3 (B) antibodies showed that stimulation does not hugely influence total protein levels. Moreover, total ERK and total STAT3 protein levels show excellent correlation with β-Actin levels in the same lanes (C and D, respectively), showing that β-Actin is a good loading control. (TIF) [file pone.0084521.s001.tif]

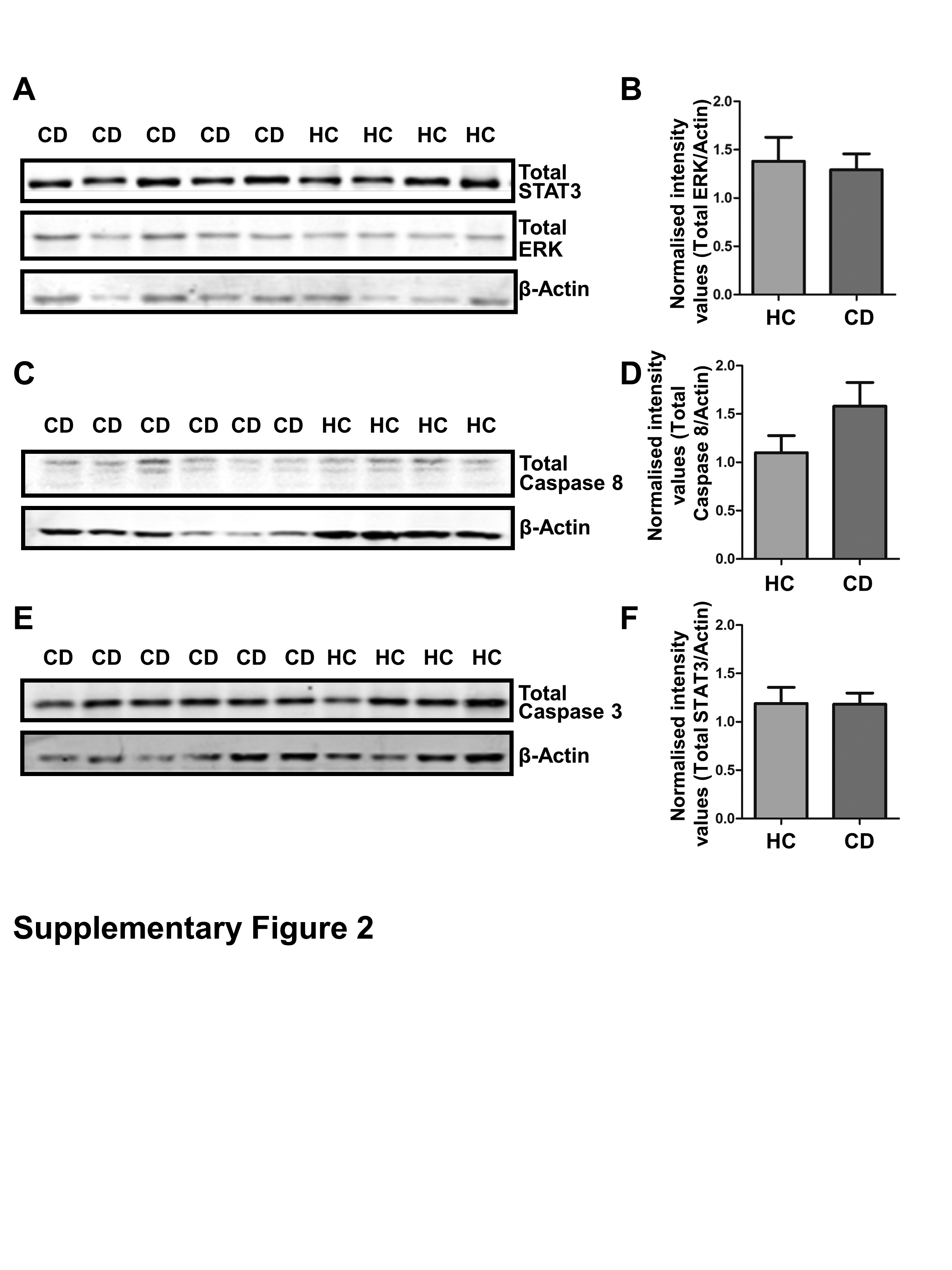

Supplement: Figure S2 — No differences in total ERK, STAT3, Caspase 3 or Caspase 8 levels between CD patients and healthy controls (HC). Unstimulated, isolated PMN from CD patients and HC were run on SDS-PAGE, and probed with antibodies against total ERK protein, total STAT3 protein (examples in panel A), total uncleaved Caspase 8 (panel C) or uncleaved Caspase 3 (examples panel E, more in main manuscript). Quantitation of blots showed no differences in total ERK levels between CD (n=18) and HC (n=16, p=0.6915, panel B). There were no differences in total STAT3 levels between CD (n=24) and HC (n=22, p=0.448, panel F). There were no differences in total uncleaved Caspase 8 levels between CD (n=16) and HC (n=16, p=0.266, panel D). Quantitation of total uncleaved Caspase 3 levels is shown in manuscript, Figure 4B. (TIF) [file pone.0084521.s002.tif]
